# The Autonomic Nervous System in Acupuncture for Gastrointestinal Dysmotility: From Anatomical Insights to Clinical Medicine

**DOI:** 10.7150/ijms.107643

**Published:** 2025-05-20

**Authors:** Na-Na Yang, Xiao-Xia Xie, Wen-Li Yan, Yi-Duo Liu, He-Xuan Wang, Liu-Xin Yang, Cun-Zhi Liu

**Affiliations:** International Acupuncture and Moxibustion Innovation Institute, School of Acupuncture-Moxibustion and Tuina, Beijing University of Chinese Medicine.

**Keywords:** gastrointestinal dysmotility, acupuncture, autonomic nervous system, acupoints, frequency and intensity

## Abstract

The expanding knowledge on the involvement of the autonomic nervous system in regulating digestive tract homeostasis is shedding new light on the therapeutic potential of acupuncture. This review aims to evaluate the effectiveness and autonomic nervous system associated mechanisms of acupuncture in gastrointestinal dysmotility in both laboratory and clinical settings, guiding future studies. Mechanistically, studies with animal models and human subjects demonstrated that acupuncture, as a non-invasive somatosensory stimulation, can help improve gastrointestinal dysmotility via directly modulating the activation of autonomic nervous system. Additionally, acupuncture has been found to suppress inflammatory response and alter the secretion of gastrointestinal hormones via different somatic autonomic reflex pathways. Importantly, the therapeutic effects of acupuncture are affected by, and even rely on, the selected acupoints, the frequency and intensity of stimulation, type of gastrointestinal disorders, and the treatment frequency. Meanwhile, clinical studies indicated that acupuncture shows promise in alleviating gastrointestinal dysmotility from the esophagus to the colon, including conditions like gastroparesis, functional dyspepsia, postoperative ileus, irritable bowel syndrome, and chronic functional constipation. We argued against a narrow classification of abdominal and hindlimb acupoints as solely activating sympathetic or vagal nerve to decrease or increase gastrointestinal motility, respectively. Lastly, incorporating new technologies will assist to reveal central autonomic network changes in response to acupuncture stimulation.

## 1. Introduction

The autonomic nervous system (ANS), characterized by its complexity and precise regulation, oversees multiple functions of the gastrointestinal tract, including motor, secretory, sensory, storage, and excretory activities. Dysfunctions in the gastrointestinal tract resulting from ANS disorders primarily manifest as dysmotility, rather than secretory abnormalities 1. This review emphasizes the innervation of the vagal and sympathetic nerve in gastrointestinal tract, with particular attention to the central origins and anatomical features of the ANS related to gastrointestinal motility. Additionally, we explore the therapeutic potential of acupuncture stimulation via ANS in addressing gastrointestinal dysmotility, spanning from bench to bedside.

## 2. Central origins and anatomical characteristics of the ANS in gastrointestinal control

### 2.1 Afferent fibers in gastrointestinal tract

**Figure 1:**
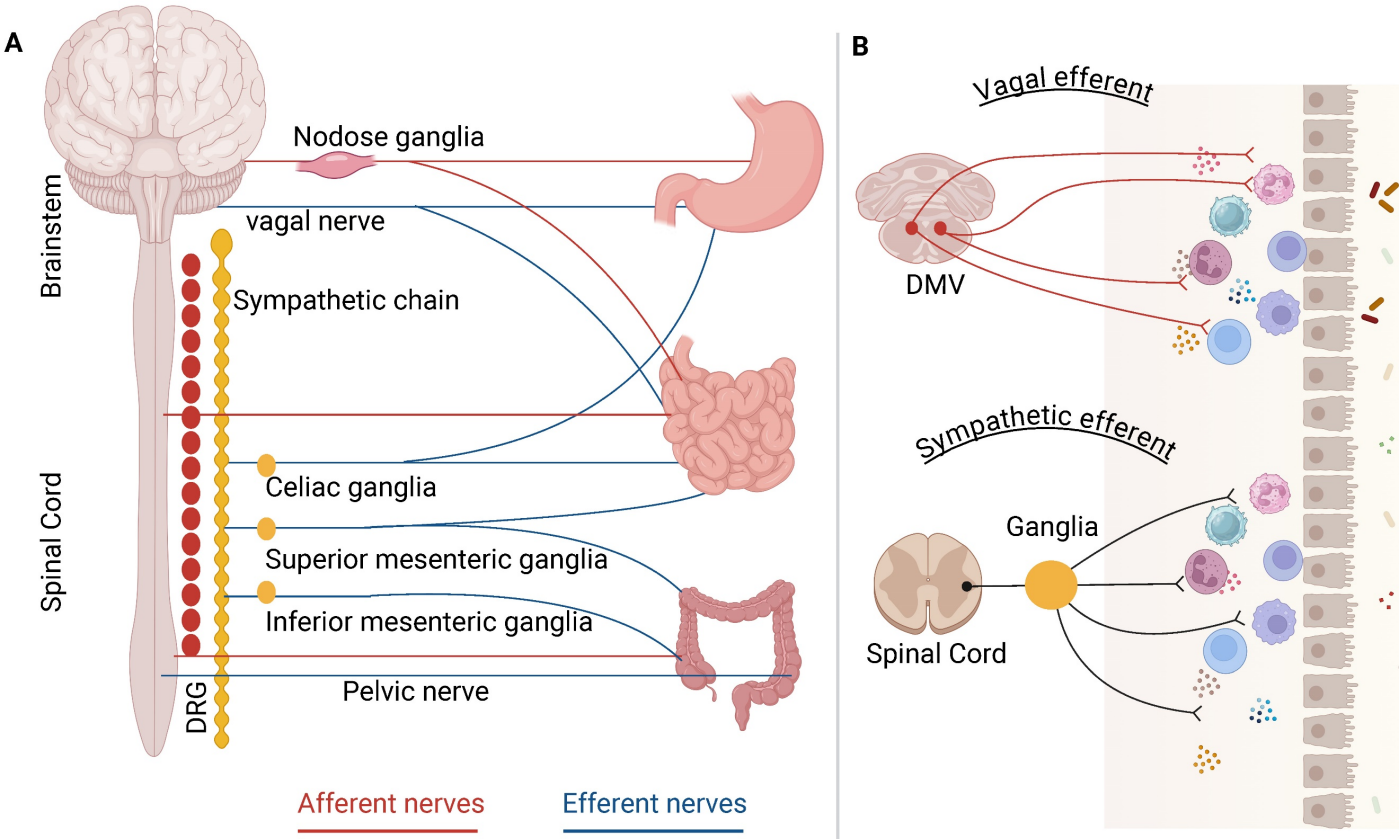
** Anatomical characteristics of the ANS in gastrointestinal control.** The left panel (A) shows extrinsic neural control, including vagal, sympathetic, and pelvic nerve. Redrawn from Figure 1A in Amanda (2021). The right panel (B) shows vagal efferent and sympathetic efferent in gastrointestinal tract.

The autonomic nerve circuits, comprising afferent and efferent signals, play crucial roles in the regulation of gastrointestinal function. A sophisticated afferent innervation transmits sensory data from the digestive system to the central nervous system (CNS), facilitating the coordination and integration of gut reflex functions and associated behavioral responses. The afferent fibers utilize two primary pathways to access the CNS: vagal afferent and spinal afferent. Vagal afferent neurons are bipolar, with their axons originating from cell bodies in the nodose ganglion (NG); one end of these axons terminates in the hindbrain, while the other extends to peripheral organs 2. Vagal afferent axons are distributed throughout the gastrointestinal tract, including stomach, duodenum, small intestine, and large intestine. Afferent signals from the gastrointestinal tract are transmitted to the nucleus tractus solitarius (NTS). Furthermore, spinal afferents are categorized into two groups: splanchnic afferent and pelvic afferent. These groups generally trace the routes of sympathetic and parasympathetic neurons that innervate to the digestive system having preganglionic cell bodies located in the spinal ganglia of thoracolumbar and sacral (Figure 1A).

Anatomical and functional distinctions can be found among the various types of sensory afferents that supply the gastrointestinal tract. The fibers of vagal afferent predominantly populate the upper gastrointestinal area, characters with the stomach having a particularly dense vagal innervation which decreases distally and becomes spares in the colon 3. In contrast, pelvic afferent is restricted to the lower part of the intestine. Innervation from splanchnic afferent extends throughout the entire gastrointestinal tract 4. Additionally, the distinct developmental trajectories of these two types of vagal afferent align with their proposed functional roles: they serve intraganglionic laminar endings, acting as mechanoreceptors that assist in coordinating rhythmic motor functions and intramuscular arrays, serving as stretch receptors that facilitate the transit of solid food 5.

### 2.2 Efferent fibers in gastrointestinal tract

The descending autonomic responses are governed by two primary nervous systems: the sympathetic and parasympathetic nervous systems, which utilize noradrenaline and acetylcholine as neurotransmitters, respectively 6. Neurocircuits involving the vagal nerve comprise distinct nuclei located within the caudal brainstem, specifically the NTS, the dorsal motor nucleus of the vagus (DMV) and the nucleus ambiguous. Neurons within the NTS process visceral sensory data, while the DMV and nucleus ambiguous serve as the sources of vagal motor axons 7. Efferent fibers that stem from the DMV engage with postganglionic neurons in target organs, ultimately influencing gastrointestinal motility and various other visceral functions (Figure 1B). The organization of DMV neurons is viscerotopically arranged 8: neurons that innervate the anterior gastric area and liver reside in the left DMV, while those innervating the posterior gastric and intestinal areas are found in the right DMV. A considerable number of DMV neurons are cholinergic releasing acetylcholine to activate nicotinic receptors on postganglionic neurons present in the relevant organs 7.

Preganglionic sympathetic nerve fibers establish synapses with postganglionic sympathetic nerve fibers within the sympathetic ganglia, which are situated in proximity to the spinal cord (Figure 1B). The projections from the prevertebral sympathetic ganglia to various regions of the gastrointestinal tract exhibit a somatotopic organization 9. The most rostral ganglia, namely the splanchnic, coeliac, and superior mesenteric ganglia, contain neurons that innervate all regions of the gastrointestinal tract, as well as the pancreas and spleen. In contrast, the inter-renal and inferior mesenteric ganglia, which are positioned more caudally, comprise neurons that innervate the distal portions of the gut, specifically the distal ileum and colon. The catecholaminergic neurons predominantly provide sympathetic input to the gut. The regulation of gastric motility by sympathetic fibers mainly requires presynaptic inhibition of vagal cholinergic inputs targeting postganglionic neurons within the enteric plexus 10.

## 3. ANS-imbalance in gastrointestinal dysmotility

Gastrointestinal dysmotility originate throughout the entire length of the gut, from the esophagus to the colon, and are characterized by symptoms such as early satiation, nausea, vomiting, abdominal pain, bloating, and other 11. Gastrointestinal dysmotility is one of the most prevalent pathological features associated with various clinical conditions, including gastroparesis, functional dyspepsia (FD), irritable bowel syndrome (IBS), postoperative ileus (POI), and chronic functional constipation (CFC). For instance, nearly every patient undergoing abdominal surgery experiences gastrointestinal dysmotility, which contributes to significant health care costs and a decline in quality of life 12. Despite the rising prevalence of gastrointestinal motility disorder as a public health concern, treatment options remain limited due to inadequate understanding of pathophysiological mechanisms 13.

While the gastrointestinal system contains inherent neural networks that enable a certain level of autonomous regulation of its activities, the brain and spinal cord provide extrinsic neural inputs 3, primarily through ANS. A notable characteristic of the ANS is that a single input can exert both positive and negative effects on gastrointestinal functions. Under physiological conditions, the sympathetic and parasympathetic nervous systems function are in a coordinated and dynamically interactive manner. The sympathetic nervous system predominantly inhibits gastrointestinal muscle activity to maintain homeostasis, whereas the parasympathetic nervous system precisely regulates the rhythmic contraction and relaxation of the stomach and intestines 3. Imbalance in the sympathetic and parasympathetic nervous systems has been linked to gastrointestinal motility issues. As a result, increasing amounts of research were conducted to investigate the role of these systems in maintaining digestive tract balance, offering new perspectives for potential therapeutic interventions 14.

## 4. Targeting the ANS for the treatment of gastrointestinal dysmotility in acupuncture treatment

Acupuncture, a treatment rooted in traditional Chinese medicine, has been empirically practiced for several millennia in the context of gastrointestinal motility. Numerous studies have explored the effectiveness of acupuncture in treating gastrointestinal dysmotility 15, 16. As a non-invasive strategy for nerve stimulation, acupuncture shows promise as a potential therapeutic approach to modulate the ANS balance and improve gastrointestinal dysmotility over the long term 17. Since the 1970s, research has indicated that stimulation through pinch or electroacupuncture (EA) in the abdominal and hindlimb areas has been shown to distinctly influence sympathetic and vagal efferent pathways related to gastric regulation 18. Further investigations revealed the mechanisms underlying the distinct effects of regional acupoints versus heterotopic acupoints 19, 20. As the use of acupuncture continues to rise, a substantial amount of research is emerging regarding mechanisms associated with the VNS.

**Figure 2:**
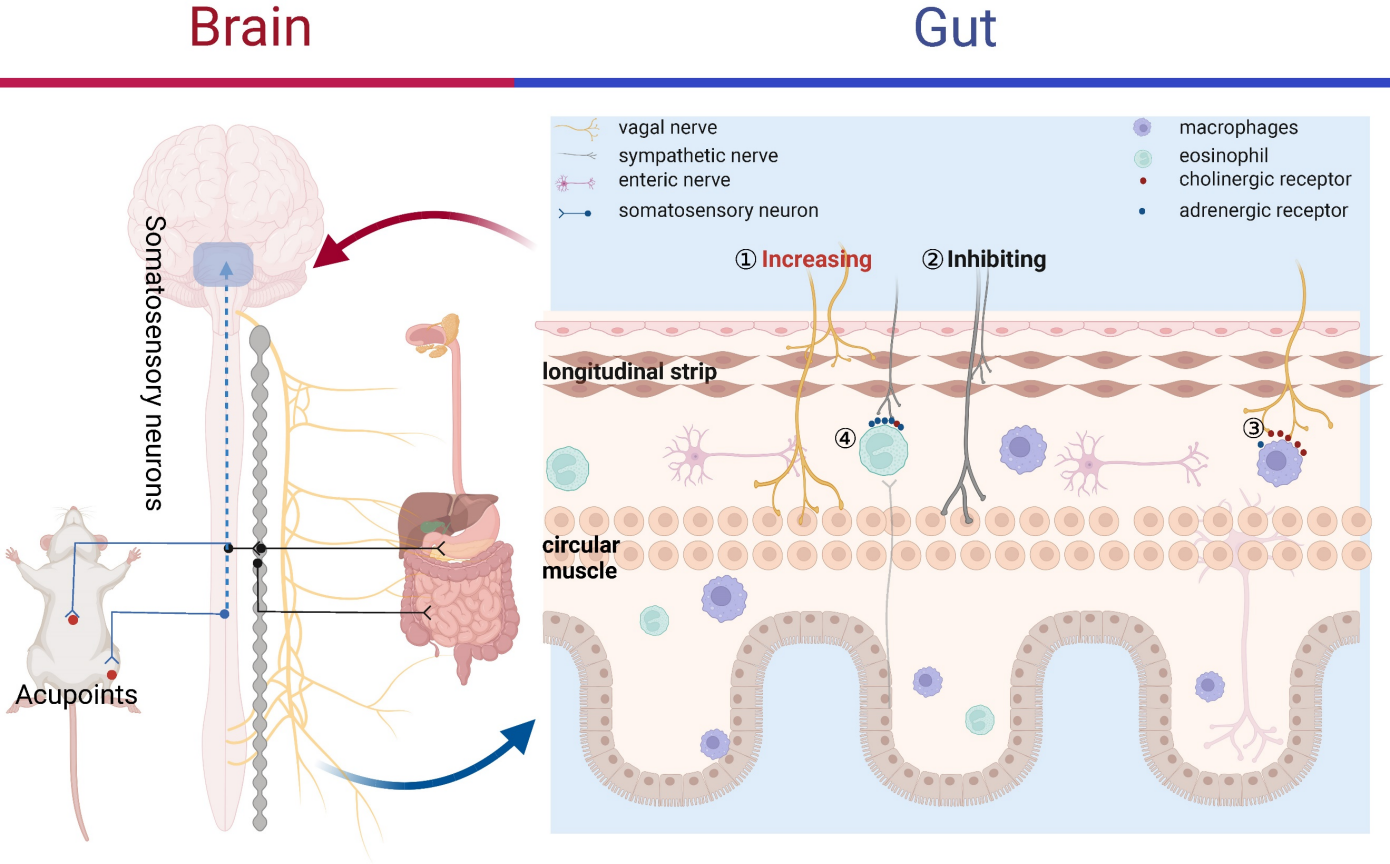
** Acupuncture improved gastrointestinal dysmotility.** Acupuncture at abdominal and hindlimb regions was reported to differently drive sympathetic versus vagal efferent pathways associated with gastrointestinal motility. Acupuncture could directly enhance vagal activity^①^ or suppress sympathetic activity^②^ to improved GI dysmotility. Acupuncture suppressed macrophages activation via vagal nerve-cholinergic receptor pathways^③^ and altered eosinophil through sympathetic-adrenergic receptor pathway^④^.

A traditional Chinese medicine theory “Acupuncture is believed to restore the balance of *Yin* and *Yang*” could be translated to “Acupuncture modulates the imbalance between the parasympathetic and sympathetic activity” in the Western medicine terminology 21. While the precise mechanisms by which acupuncture improves conditions related to gastrointestinal motility diseases remain partly understood, both fundamental and clinical research evidence appears to endorse the following processes: acupuncture stimulates somatic and peripheral nerves, which transits an afferent signal to the brain and spinal cord, leading to an increased vagal activity or a reduction in sympathetic efferent output to the digestive system (Figure 2), ultimately enhancing motility 15, 21. Acting as a two-way connector between the brain and the gut, the ANS is essential for maintaining gastrointestinal homeostasis. It influences various functions, including motility, immune response, acid secretion, and the release of enteric hormones, through its roles in sensing and modulation. The imbalance of the parasympathetic and sympathetic nervous system was observed in a variety of gastrointestinal disorders, therefore, restoring ANS may be a therapeutic target.

### 4.1 Acupuncture improved gastrointestinal dysmotility via modulating ANS activity

Parasympathetic nerve exerts a predominantly excitatory effect upon gastrointestinal muscle, orchestrating the intricate interplay between central and peripheral neural control mechanisms. As an indicator of autonomic imbalance, reduced vagal tone evaluated assessing by heart rate variability (HRV) contributes to gastrointestinal impairment. Compared with healthy participants, FD patients were accompanied by a lower vagal tone 22. Similarly, compared with type 1 diabetic patients following peripheral neuropathy, those patients with low cardiac vagal tone had more serious impairing of gastric motility and colonic transit 23. These results indicated that low vagal tone was known to contribute to gastrointestinal dysmotility.

Restoring the typical vagal tone might be significant in the medical treatment of these gastrointestinal disorders. VNS, either invasive or non-invasive, opens therapeutic avenues. A clinical study recruited ten gastroparetic patient's refractory to standard medical therapy to explore the effects of invasive gastric electrical stimulation (GES) on autonomic function 24. This study demonstrated that GES had potential to influence CNS control mechanisms related to nausea and vomiting via activation of vagal afferent pathway, and then increased vagal efferent tone to mitigate gastric sensitivity, all of which ameliorated postprandial gastric accommodation. Meanwhile, the sacral parasympathetic nucleus innervates the left colon and rectum, and at present, sacral nerve stimulation is the sole approved neuromodulation approach for managing fecal incontinence. Likewise, chewing gum 25 and sham feeding 26 as non-invasive cephalic stimulation, were sufficient to up-regulate vagal tone and then normalized meal-induced antral dysmotility in FD patients or reduced time to flatus and time to passage of bowel motion in POI patients. Conversely, patients with gastrointestinal dysmotility were dissatisfied with invasive VNS due to additional surgery and side effects or unwilling to try non-invasive VNS such as chewing gum, due to limited efficacy 25.

Acupuncture, a surface nerve stimulation, had potential to enhance vagal activity with multi-benefits, such as the excellent safety record, more accessible and facilitating, and well-tolerated (Figure 2). In the clinical study, *Neiguan* (P6) acupoints was able to improve nausea and vomiting, which were associated with the increasing vagal tone assessed by HRV in the healthy subjects 27. Animal studies such as that conducted by *Yi Guo* have also shown that gentle (2 Hz) manual acupuncture increased motility amplitude of the stomach via vagal nerve, but not strong (4 Hz) manual acupuncture 28. EA at ST36 restored rectal distension-induced impairment of motor by enhancing vagal activity in gastroparesis of dogs 29, 30. Meanwhile, EA also improved gastric dysrhythmia, delayed gastrointestinal transit, and impaired accommodation mainly through vagal nerve in diabetic rats 31. Likewise, 2 Hz stimulation at ST36 partly shorted the recovery time by directly activating the vagal nerve in POI mice, but not regulating local gastrointestinal inflammation 32. Further mechanism studies indicate that acupuncture stimulation promoted efferent vagal nerve activity and increased gastric motility via inhibiting gamma-aminobutyric acid (GABA) transmission in DMV 33.

Reciprocally, the sympathetic nervous system provided a primarily inhibitory effect on gastrointestinal muscles (Figure 2). Concurrent with the increase in vagal activity, it is not unusual for acupuncture to reduce sympathetic activity. Back in 1997, *K Yazawa* et al found that acupuncture at *Ximen* (P4) lowered the heart rate, an effect that was countered by the administration of atropine and propranolol. This suggests that the impact of acupuncture on heart rate is due to a reciprocal interaction within the ANS, characterized by an increase in vagal activity and a reduction in sympathetic activity 34. Besides, acupuncture at *Sishencong* points also improved cardiac vagal tone while inhibiting sympathetic activity in humans 35. In constipation model rat, EA at ST36 shorten distal colon transit time, increased small intestinal transit and gastric emptying via increasing vagal tone and decreasing sympathetic activity 36. While the extensive amount of information currently highlights the intricate roles of vagal nerve and sympathetic nerve in controlling gastrointestinal motility, there remains a lack of data concerning the impact of acupuncture stimulation on sympathetic activity specifically. Therefore, the precise mechanism through which acupuncture may reduce sympathetic activity is unclear and future studies are needed.

### 4.2 Acupuncture improved gastrointestinal dysmotility via ANS suppressing inflammation

Recently, investigations indicated that immune-mediated pathophysiological and molecular events within the gastrointestinal tract are likely contributing factors 13. Multi-immune cells are residing in gastrointestinal tract, such as macrophages, eosinophils, mast cells, and T cells. When trigger, they can lead to the production of inflammatory cytokines, subsequently increasing vascular permeability and altering smooth muscle contraction, resulting in visceral hypersensitivity and dysmotility 37, 38. Cytokine synthesis and release including TNF-α, IL-1β, IL-6, and others, are essential components of the innate immune system. However, inappropriate or excessive production of these cytokine can lead to damage in distant organs 39. Therefore, it is generally accepted that low-grade inflammation and innate immunity are central to the pathogenesis of gastrointestinal dysmotility 40.

The vagal nerve serves as a crucial initial line of innate defense against infection and inflammation while contributing to the restoration of homeostasis within the body 41. Vagal afferent fibers sense peripheral low-level cytokines via the ascending fibers, especially, vagal nerve was equipped with IL-1β receptor to convey the information to the brain 42, 43. Pro-inflammatory cytokines activated vagal afferent, who excited hypothalamic-pituitary-adrenal axis (HPA) in turn suppressed gastrointestinal inflammatory response via increasing the release of cortisol by the adrenal glands 44. Given its position as a key element of the vagal nerve in peripheral inflammatory response, its activation seems to be a good therapeutic target for gastrointestinal dysmotility.

The anti-inflammatory function of vagal efferent fibers is mediated through two pathways: splenic anti-inflammatory pathway and cholinergic anti-inflammatory pathway (CAIP). The spleen was the major source of cytokines in systemic inflammation such as sepsis 45, but not in local inflammation such as POI, IBS, and other more subtle inflammatory response. Therefore, the CAIP is a more important strategy in the treatment of various gastrointestinal dysmotility diseases (Figure 2). The link of acetylcholine (Ach) releasing from the distal end of the vagal nerve and α7-nicotinic Ach receptor (α7nAchR) in immune cells had the potential to suppress the production of pro-inflammatory cytokines, but not influence anti-inflammatory cytokines, such as IL-10 46. Against this backdrop, CAIP offered the host a rapid, targeted, and confined method for regulating the immune response and avoiding excessive inflammation.

Acupuncture remarkably suppressed pro-inflammatory cytokine production via activating CAIP and then improved gastrointestinal dysmotility 47. Based on the CAIP and the initial vagal nerve data, we aimed to explore the processes through which stimulation of ST36 via EA reduced inflammation in POI mice 48. In this study, we demonstrated that EA activated the α7nAchR-mediated Janus kinase 2/signal transducer and activator of transcription 3 (JAK2/STAT3) signaling pathway in macrophages which reduced the production of TNF-α and IL-6, and then remarkedly promoted gastrointestinal motility. Furthermore, the protective role of EA was negated by conducting cervical vagotomy, sub-diaphragmatic vagotomy or by microinjecting muscimol (agonist of GABA_A_ receptor) into the DMV. This suggests that acupuncture enhanced vagal efferent activity by reducing the expression of GABA_A_ receptors in the DMV, thereby alleviating local gastrointestinal inflammation. Furthermore, the distribution patterns of PROKR2^Cre^ nerve fibers were able to predict whether EA will produce anti-inflammatory effects via vagal nerve or not 19. The excessive production of pro-inflammatory cytokines and activation of the immune system contributed to the development of colitis 49. *Chen* et al. 50 found that VNS and EA were shown to suppress production of multi pro-inflammatory cytokines via increasing vagal activity while reducing sympathetic activity, thereby improving both macroscopic and microscopic damage in colitis rats. Additionally, VNS plus EA provided not only yielded considerable anti-inflammation benefits but also showcased a synergistic impact of acupuncture. Likewise, EA at ST36 enhanced gastric dysrhythmia and promoted gastric emptying through the vagal pathway, which involves the inflammatory cytokine IL-6 in rats suffering from burns 51. Therefore, acupuncture has potential to suppress local inflammatory via vagal verve mediated CAIP, aiming to ameliorate gastrointestinal motility.

Research has primarily focused on the vagal nerve mediated CAIP, while there is significantly less data regarding the impact of the sympathetic system on the inflammatory response. Sympathetic nerve also has an important immunosuppressive role through adrenoceptors which were expressed on the surface of lymphocytes, such as eosinophils, macrophages, T and B cells. Consequently, these immune cells and their corresponding cytokines will react to the sympathetic nerve. Previous research found that central injection of interferon-alpha in rats could excite the splenic sympathetic nerve, in turn suppress the cytotoxicity of natural killer cells via β-adrenergic mechanisms, indicating the key role of sympathetic in immunosuppression 52. Besides, global sympathetic inhibition using reserpine produced more TNF-α in sepsis model, which was reversed by administration of β-adrenoceptor agonists 53. Therefore, it would be beneficial to outline the function of sympathetic nerve in anti-inflammation of acupuncture. Lee et al. 54 revealed that EA at ST36 with low-frequency (1Hz) significantly restrained zymosan-induced leukocytes migrating by activating sympathetic nervous system. Meanwhile, in this study, chemical sympathetic denervation or systemic propranolol administration was able to inhibit the anti-inflammatory effects of EA, but adrenalectomy did not. These findings indicated that EA exhibited anti-inflammatory properties by stimulating sympathetic nerve resulting in the releasing of catecholamines from post-ganglionic nerve terminals, which link with β-adrenoceptors on immune cells, consequently inhibiting their migration (Figure 2). Recently, a novel study demonstrated that for systemic inflammation prevention, the spinal-sympathetic axis evoked by 3 mA EA at the abdominal ST25 acupoint had potential to inhibit splenic inflammation 20. Despite of sympathetic in immunosuppression discussed above, acupuncture suppressed inflammatory response via sympathetic nerve in gastrointestinal dysmotility diseases remain fairly limited, more studies are needed in this field.

### 4.3 Acupuncture improved gastrointestinal dysmotility via ANS altering hormones

**Figure 3:**
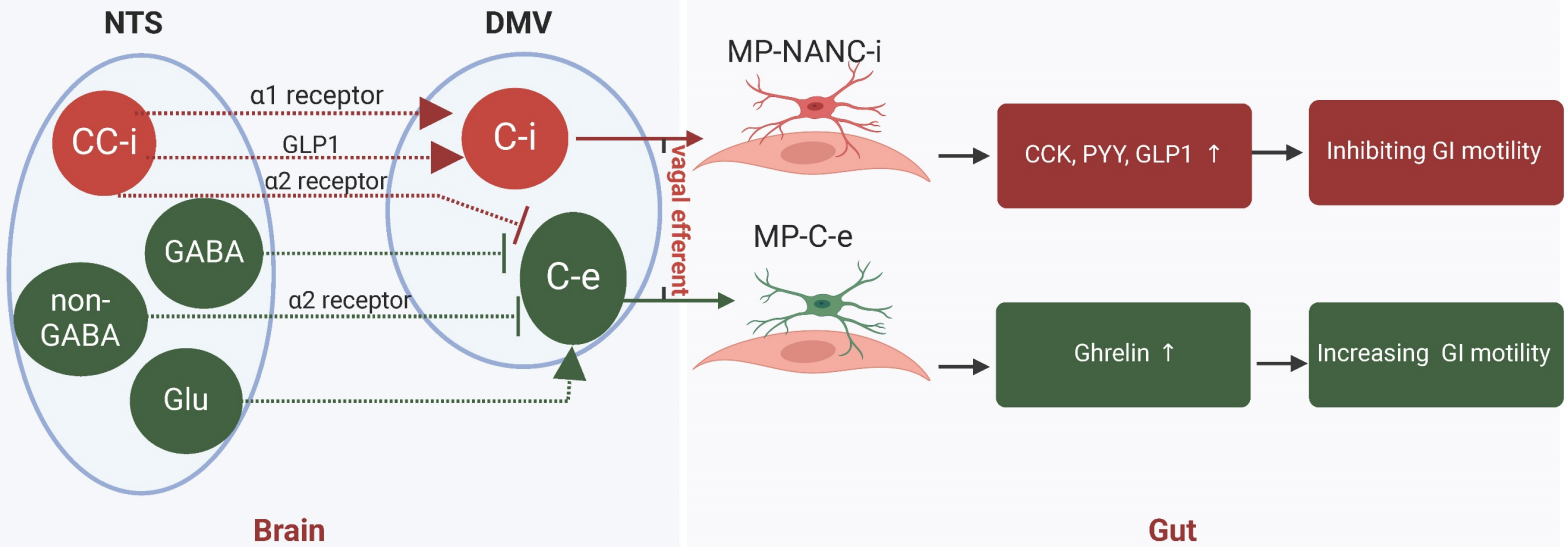
** Vagal verve altered hormones to improved gastrointestinal dysmotility.** Vagal nerve exerted both inhibitory (GIVMC) and excitatory (GEVMC) effects in hormones secreting. GEVMC: DMV-C-e neurons received strong inhibitory input from NTS-GABA neurons and NTS-CC-e neurons. and excitatory input from NTS-Glu neurons. When GEVMC trigged, hormones ghrelin, motilin and other “accelerating” hormones were augmented to hasten GI motility. GIVMC: The DMV-C-i neurons received excitatory input directory from NTS-CC-i neurons via the α1 receptor and GLP-1. Meanwhile, NTS-CC-i neurons also send inhibitory input to DMV-C-e neurons via α2 receptor. When GIVMC trigged, CCK, GLP-1, PYY, and other “braking” hormones were enhanced to inhibit GI motility. GEVMC, gastric excitatory vagal circuit; GIVMC, gastric inhibitory vagal circuit; NTS, nucleus tractus solitarius; DMV, dorsal motor nucleus of vagus; CC, catecholaminergic; i, inhibitory; e, excitatory; CCK, cholecystokinin; GLP-1, glucagon-like peptide-1; PYY, peptide YY; MP, myenteric plexus; NANC, non-adrenergic, non-cholinergic.

Historically, it was believed that the vagal nerve and sympathetic nerve played opposing roles in regulating gastrointestinal motility, with the former being excitatory and the latter inhibitory. Nevertheless, recent research indicated that the sympathetic nerve does not significantly influence the secretion of gastrointestinal hormones, whereas the vagal nerve is responsible for both inhibitory effects (via the gastric inhibitory vagal circuit, GIVMC) and excitatory effects (through the gastric excitatory vagal circuit, GEVMC) on hormone secretion 55, 56. GEVMC included excitatory preganglionic and postganglionic cholinergic neurons in the DMV (DMV-C-e) and myenteric plexus (MP-C-e). When GEVMC trigged, hormones ghrelin, motilin and other “accelerating” hormones were augmented to hasten gastrointestinal motility. In contrast, GIVMC contented preganglionic cholinergic neurons in the DMV (DMV-C-i) and postganglionic inhibitory neurons in myenteric plexus (MP-NANC-i). When GIVMC was trigged, cholecystokinin (CCK), glucagon-like peptide-1 (GLP-1), peptide YY (PYY), and other “braking” hormones were enhanced to inhibit gastrointestinal motility 57. Therefore, it would be more informative to explore the role of acupuncture on gastrointestinal peptides via vagal nerve in gastrointestinal dysmotility (Figure 3).

A large and growing body of clinical and animal literature has investigated the relationship between acupuncture, vagal nerve, motility, and gastrointestinal hormones. In diabetic patients with gastric dysrhythmia, electrical stimulation at ST36 could enhance the regularity of gastric myoelectrical activity through increasing human pancreatic polypeptide in serum 58. Our previous randomized clinical trial also found that 4-week acupuncture was able to increase the serum ghrelin concentration, which is positive correlation with the improvement of FD symptoms 59. A small but convincing animal study also found that acupuncture at ST36 for 5 days could down-regulate the concentration of vasoactive intestinal peptide (VIP) and up-regulate motilin, ghrelin and gastrin, aiming to enhance the small intestinal motility 60. Studies such as that conduct by *Chen* 61 has shown that EA at ST36 alleviated dyspeptic symptoms and enhanced gastric motility by boosting vagal efferent activity while inhibiting the anorexigenic hormones PYY and GLP-1 in rats with chemotherapy-induced dyspepsia syndrome. Particularly, *He* et al. 62 found that EA at* Zhongwan* (RN12) and* Weishu* (BL21) not only increased neuron-activating frequency in DMV, but also enhanced the levels of gastrointestinal hormones, such as motilin and gastrin, and their respective receptors in the paraventricular hypothalamic nucleus (PVN) and gastric antrum, and then regulated gastric motility. Both gastrointestinal hormones and their receptors play crucial roles in the regulation of gastric motility. Clinically, serotonin receptor agonists have been employed to treat dyspepsia symptoms, and other hormone receptor agonists or antagonists are currently under development 63. However, while gastrointestinal hormones have been extensively studied, there is a paucity of information regarding the impact of acupuncture on their receptors. Consequently, further investigation into the modulation of hormone receptors by acupuncture is warranted.

## 5. The characteristics of acupuncture on regulating ANS in gastrointestinal motility

Conceptually, it is thought that acupuncture encourages the body's meridians, which are channels that carry energy, in order to address imbalances and promote well-being. As mentioned previously, a considerable number of studies have investigated the efficacy and mechanisms of acupuncture in gastrointestinal dysmotility based on ANS-associated multi-pathway 20, 29, 48. The effectiveness of acupuncture treatment is affected by, and can even rely on, the selected acupoints, the frequency and intensity of stimulation, as well as various other factors.

### 5.1 Abdominal acupoints and hindlimb acupoints

The acupoints located in the abdomen, like ST25, and those in the hindlimbs, such as ST36, were among the most frequently utilized acupoints for addressing gastrointestinal dysmotility 64. To some degree, the targeted stimulation of particular acupoints may establish a contemporary neuroanatomical foundation for connecting somatic tissue stimulation to the regulation of internal organ function, a role historically believed to be performed by meridian channels, which are yet to be physically identified 20. Several investigations have indicated that pathways related to vagal and sympathetic responses, which are involved in gastric motility regulation, can be selectively activated through limb and abdominal acupoints, respectively 65, 66. These somatosensory autonomic pathways earlier findings by Sato in anesthetized rats 65 showed that the sympathetic efferent nerve activation mediated the inhibitory gastric response resulting from abdominal stimulation, with its reflex center located in the spinal cord. In contrast, the excitatory gastric response elicited by hindpaw stimulation was governed by the activation of the vagal efferent nerve, which necessitated the involvement of the brain as its reflex center. This theory is gradually popular and accepted in gastrointestinal diseases and was proved by several studies 20, 48, 67.

However, recent studies demonstrated that acupuncture on gastrointestinal motility is still controversial, indicating that abdominal acupoint and hindlimb acupoints were not narrowly defined as activating sympathetic or vagal nerve to decrease or increase gastrointestinal motility, respectively. On the one hand, acupuncture at hindlimb acupoints induced dual effects, either stimulatory or inhibitory, on gastric motility via vagal efferent in conscious rats, which were dependent on the initial gastric activity. This study found that the stimulatory effects of acupuncture at ST36 were observed only in no strong contraction rats, meanwhile, the inhibitory effects were mainly observed in showing contraction rats 68. Likewise, acupuncture at hindlimb acupoints also induced dual effects in human study, depending on the initial gastric activity.

On the other hand, abdominal acupoints also have the potential to enhance gastrointestinal motility via stimulating the vagal pathway or inhibiting sympathetic pathway. Previously significant focus of abdominal acupoints have been placed on the level of spinal cord. In 2015, the initial research demonstrating the acupuncture signals from the gastric *Shu* (RN12) and *Mu* (BL21) points converging in higher nerve centers was conducted 62. This study found that the signals induced by EA at RN12 (back acupoint) and BL21 (abdominal) gathered not only in spinal cord but also evoked a “targeted convergence” in DMV, PVN, and hypothalamus, and then to regulate gastric motility, emphasizing that EA at abdominal and back acupoints ameliorated gastrointestinal motility via PVN-DMV-vagal-gastric pathway. Meanwhile, there is a large volume of published studies describing the inhibitory effect of acupuncture in sympathetic nerve activity in heart-associated diseases 69-71, demonstrating that acupuncture is able to inhibit sympathetic activity via high-threshold supraspinal pathways, but needs to be investigated further in gastrointestinal diseases. Consequently, it appears that the influence of acupuncture or electroacupuncture on gastrointestinal motility relies on several factors, including specific acupoints and the baseline motor activity.

### 5.2 Acupuncture dose

Acupuncture drives vagal and sympathetic nervous system via specific somatosensory autonomic pathways, indicating that the therapeutic effects of acupuncture were also influenced by somatosensory neurons. Activation of distinct sensory afferents has potential to induce specific autonomic reflexes. Particularly, somatosensory neurons are thought to be heterogeneous, with different axon diameters and myelination degrees, all of which impacted activation of these sensory afferents in response to stimulation 72. Against this backdrop, different stimulatory frequencies and intensities will excite different sensory afferent.

Due to the larger diameter axon having lower threshold intensity for conduction block, low-intensity stimulation is able to activate larger myelinated fibers (such as Aδ and Aβ fibers) while high-intensity has ability to excite thinly myelinated and unmyelinated fibers (such as C fibers) 73. Ulloa and colleagues 74 verified that EA at ST36 suppressed systemic inflammation via a vagal-adrenal axis, which is voltage-dependent. Similarly, Ma and colleagues 20 also found that low intensity (0.5mA) EA stimulation at ST36 had ability to drive vagal anti-inflammatory pathway, whereas, high-intensity (3mA) at ST36 or ST25 was needed to drive sympathetic anti-inflammatory pathway. Meanwhile, a separate study examined how only acupuncture stimulation that exceeds the threshold of Aδ and/or C afferent fibers can significantly influence gastrointestinal motility 75. In this study, LI11 in forelimb, ST13 in upper-breast, ST21 and CV6 in abdomen, and ST36 in hindlimb were studied. Acupuncture with non-noxious stimulation (0.8-T_Aδ_, about 1.42mA) did not produce any significant influence on gastrointestinal motility. Compared with acupuncture with slight-noxious stimulation (2-T_Aδ_, about 3.62mA), strong-noxious acupuncture stimulation (1.5-T_C_, about 7.68mA) at LI 11, ST13, and ST36 elicited a strong excitatory gastric motility, meanwhile, the acupuncture stimulation at ST21and CV6 brought a strong inhibition on motility.

Acupuncture with different stimulatory frequencies at same acupoints had different therapeutic effects on gastrointestinal motility and inflammation. Our previous study in POI model mice found that EA at ST36 using either 10 Hz or 30 Hz markedly enhanced gastrointestinal motility and reduced peripheral inflammation; in contrast, applying ST36 stimulation at 2 Hz or 100 Hz showed no effect 76. Similarly, another study investigated the effects of EA frequencies (2Hz,100Hz, and alternate 2/100Hz) on ST36 and ST25 77. This research demonstrated that compared with 100Hz or alternate 2/100Hz, EA at ST36 with 2Hz had the strongest excitatory effect on NTS neurons, meanwhile, compared with 2Hz or alternate 2/100Hz, EA at ST25 with 100Hz had the greatest inhibitory effect on gastrointestinal motility. Besides, the effects of manual acupuncture were also affected by frequency. Compared with 1Hz, 3Hz, and 4 Hz, 2Hz manual acupuncture exhibited the most effectiveness in increasing gastric motility 28.

Like medications, a relationship between the frequency of acupuncture treatments and their effectiveness exists, although it still requires further confirmation. Our previously randomized clinical trial with 60 FD-patients found that compared to once a week, acupuncture with 3 sessions per week was more effective to improve symptoms and quality of life among patients, accompanied by a long-term therapy 78. This observation supported the hypothesis that to some degree, the more treatment times were, the better effective get, but few data addressing this hypothesis in gastrointestinal dysmotility.

As mentioned previously, the roles of frequency, intensity, acupoint, and treatment times are pivotal to achieving optimal therapeutic effects of acupuncture. Consequently, prior to determining whether acupuncture is a successful clinical approach, it is advisable to conduct additional systematic clinical investigations focusing on the manipulation of parameters, informed by research that indicates effective combinations in both human and animal experimental models of gastrointestinal disorders.

### 5.3 Type of gastrointestinal diseases

A better comprehension of the pathophysiological mechanisms underlying gastrointestinal conditions will lead to new non-drug and medication-based treatments. Gastrointestinal dysmotility are diverse, involving various pathophysiological mechanisms that contribute to different patterns of disease. Therefore, it may be more plausible and effective to select stimulation acupoint or stimulation parameters depending on pathophysiology mechanisms of different gastrointestinal diseases, rather than aiming at alleviating symptoms. While structural disease serves as an exclusion criterion for FD, recent research has shown that low-grade immune activation, particularly the proliferation of activated eosinophils, may have resulted from and could also contribute to alterations in duodenal permeability 79. By contrast, it is widely acknowledged that the resident macrophage population plays a more crucial role in POI 80. Notably, the prevalence of adrenergic and cholinergic receptors varies based on different leukocyte subsets. Specifically, granulocytes (such as eosinophils) possess a high concentration of adrenergic receptors and a low concentration of cholinergic receptors on their surfaces, while lymphocytes (including macrophages) have a high concentration of cholinergic receptors and a low concentration of adrenergic receptors present on their surfaces 81 (Figure 2). These results indicated that acupuncture at hindlimb region with low-intensity and low-frequency may be more effective to suppressed macrophages activation via vagal nerve-cholinergic receptor pathways, and then improved motility of POI; acupuncture at hindlimb and abdominal region with high-intensity and high-frequency may be more suitable to altered eosinophil through sympathetic-adrenergic receptor pathway, aiming to ameliorate FD symptoms.

The therapeutic efficacy of acupuncture is significantly influenced by factors such as the selection of acupoints, the dosage of acupuncture, and the specific type of disease being treated. It is imperative that the selection of acupoints is customized to address the particular gastrointestinal disorder in question. Moreover, the acupuncture dosage should be determined based on the characteristics of the acupoints and the underlying pathophysiology of the condition to achieve optimal therapeutic outcomes. Additionally, interactions among various components of the acupuncture treatment may result in a clinical effect that surpasses the additive effects of the individual elements. Considering these factors is crucial for the design and interpretation of clinical trials involving acupuncture, as they have substantial implications for the validity and reliability of the findings.

## 6. Clinical evidence for acupuncture on upper gastrointestinal motility disorder

### 6.1 Gastroparesis

Gastroparesis is defined by delayed gastric emptying without any mechanical obstruction and identification of clinical symptoms of gastrointestinal dysmotility 82. The condition is typically categorized into two main subgroups: idiopathic gastroparesis (IG), characterized by early satiety and upper abdominal pain, and diabetic gastroparesis (DG), marked by severe retching. However, all subgroups had documentation of delayed gastric emptying 83. Metoclopramide, a dopamine D2-receptor antagonist, is currently the sole medication approved by the US FDA for gastroparesis, but only for a maximum duration of 12-week 84. Furthermore, gastric electrical stimulation has also received FDA approval under a humanitarian device exemption for patients with refractory DG or IG 85. However, the FDA issued a black-box warning for metoclopramide because of possible side effects, including tardive dyskinesia. While antiemetics may relieve nausea and vomiting, their efficacy specifically in gastroparesis has not been extensively studied 84. Given the significant impact of gastroparesis and the limitations of available treatments, there is a pressing need for new strategies to improve the clinical symptoms of gastrointestinal dysmotility.

The efficacy of acupuncture in gastroparesis patients was assessed by three systematic reviews and meta-analyses (Table S1). A meta-analysis conducted in 2013, which involved 14 randomized controlled trials (RCTs) with 948 diabetic gastroparesis patients, showed that acupuncture was more effective than domperidone in improving dyspeptic symptoms, such as nausea, vomiting, loss of appetite, and bloating with no heterogeneity 86. Likewise, another meta-analysis from 2014, comprising 7 RCTs with 730 postoperative gastroparesis patients, found that neither acupuncture alone nor acupuncture in combination with medication demonstrated a markedly greater total effective rate compared to standard care or medication alone 87. Reciprocally, a meta-analysis from 2018, involving 32 studies and 2601 participants, showed that although acupuncture might have potential to improve gastroparesis symptoms, the evidence was of very low quality 88. This review highlighted the uncertainty surrounding the effects of acupuncture in gastroparesis, emphasizing that no definitive conclusions could be drawn.

Acupuncture, as an alternative therapy, has shown short-term efficacy in the improvement of symptoms and gastric emptying on gastroparesis, especially in DG patients 89. A single-blinded, randomized pilot study found that compared with sham EA treatment, short-term EA treatment effectively reduced the dyspeptic symptoms of DG and accelerated solid gastric emptying, particularly, this improvement was maintained 2 weeks after the end of the trial 90. Similarly, some RCTs and clinical observation also found that acupuncture did give well therapeutic effects on gastroparesis, which were associated with the increasing vagal activity and the enhancement of the regularity of gastric myoelectrical activity 58, 91, 92. Compared with domperidone alone, acupuncture add domperidone treatment decreased the scores of all cardinal symptoms of the Gastroparesis Cardinal Symptom Index (GCSI), increased scores of the Satisfaction with Life Scale (SWLS) and the social functioning domain of the Short-Form (SF-36) 93, indicating that acupuncture combined with medicine may be more effective than medicine alone. Meanwhile, a multi-center RCT with 99 patients found that compared with stimulation at *Neiguan* (PC6) plus *Zusanli* (ST36) or ST36 plus non-acupoint, acupuncture at *Zhongwan* (CV12) plus ST36 was more effective in decreasing GCSI scores and each symptom-score including early fullness, stomach bloating, and feeling of fullness after a meal 94, indicating that the efficacy of acupuncture in gastroparesis was influenced by the selection and number of acupoints.

Besides, FD-symptoms were similar to those of gastroparesis, which was difficult to totally separate in clinical practice 95. The therapeutic effect of acupuncture in FD has been well investigated by several mate-analyses 96-101 and various high-quality RCTs 102-104, which were reviewed by writer in detail elsewhere 79.

### 6.2 Postoperative ileus

POI is characterized by a transient impairment of gastrointestinal motility after abdominal surgery 105. It is important to highlight that POI represents the primary reason for extended hospitalizations, with total annual costs—both direct and indirect—surpassing $1.5 billion in the United States 80. Prokinetics are commonly employed in clinical settings following surgery to alleviate symptoms 105. However, prokinetics are hampered by limited efficacy and potential side effects. Considering the significant human and financial toll, there is an urgent need for more effective and cost-efficient treatments.

The effectiveness of acupuncture in POI was first review in cancer patients and found that the therapeutic effects of acupuncture shown on different cancer-related aspects, including postoperative urinary retention, quality of life, postoperative gastrointestinal dysfunction, and prevention of prolonged postoperative ileus 106. Another meta-analysis with 15 high-quality RCTs involving 965 participants revealed that acupuncture could shorten the duration until the first flatus, defecation, bowel sound recovery, and initiation of oral feeding, while also decreasing the length of hospital stays. This evidence suggests that acupuncture is a safe and effective intervention for postoperative ileus (POI) following abdominal surgeries, including laparoscopic procedures 107. Of note, limb acupoints including ST36, *Shangjuxu* (ST37), and *Xiajuxu* (ST39), were more commonly used in POI patients 108. Similarly, a systematic review and meta-analysis with thirty RCTs involving 2976 patients demonstrated that ST36 acupoint injections with different agents may have a preventive effect on POI 109. A subgroup analysis conducted in a systematic review and meta-analysis indicated that manual acupuncture was effective for the time until the first occurrence of flatus or defecation, while EA was more effective in shortening the duration of hospital stay 110 (Table S2).

Several RCTs have shown that acupuncture had therapeutic effects in improving the symptoms of POI including pain and dysmotility, ameliorating the quality of life, and reducing length of hospital stay 111-115. A high-quality prospective study recruited 165 POI patients who were assigned randomly to EA group, sham acupuncture group, once daily from postoperative days 1-4, or no acupuncture group. This study found that compared with no or sham acupuncture group, EA had a shorter time to defecation or ambulation, and was more effective in reducing postoperative analgesic requirement 113. Several small yet fairly persuasive studies examined the impact of acupuncture on alleviating symptoms in patients with POI 111, 112. *Chae* et al 112 found that in contrast to sham acupuncture, the use of acupuncture resulted in a notably lower number of residual Sitz markers within the small intestine on the third and fifth postoperative days. This suggests that acupuncture might shorten the duration of POI following gastric surgery and could represent an effective treatment option in protocols aimed at enhancing recovery after surgical procedures.

## 7. Clinical evidence for acupuncture on lower gastrointestinal motility disorder

### 7.1 Irritable bowel syndrome

IBS is characterized by symptoms such as abdominal discomfort or pain and changes in bowel habits, occurring without any underlying diseases that could explain these symptoms 116. IBS affected 7% to 21% of the general population, and substantially reduced health-related quality of life and work productivity 117. IBS is divided into three subtypes: IBS with diarrhea (IBS-D), IBS with constipation (IBS-C), and IBS with a mixed bowel pattern (IBS-M). Patients may transition between various subtypes of IBS over time, with the most frequent shift being from IBS-C or IBS-D to IBS-M; less frequently, individuals switch between IBS-C and IBS-D 118. Historically, the primary treatments for IBS consisted of over-the-counter drugs which effectively improved bowel habits with an excellent safety record and widespread availability, for example, loperamide and probiotics for diarrhea or fiber supplements and laxatives for constipation. Nevertheless, non-prescription drugs provide minimal advantages for general IBS symptoms or abdominal issues like discomfort and bloating. Meanwhile, some of the drugs have been withdrawn from the market because of side effects and high relapse rates 119.

Acupuncture provided an alternative or adjunctive therapy for IBS patients which was supported by several systemic reviews and meta-analyses (Table S3). A systematic review and meta-analysis encompassing thirty-one studies involving 3,234 patients revealed that, in comparison to medication, acupuncture demonstrated greater efficacy in promoting weekly bowel movements, total symptoms score, and IBS-D symptom severity score, indicating that acupuncture treatment improved the global-clinical effectiveness of IBS-D 120. Similarly, a review with five RCTs (449 participants) also found that 84% of individuals receiving acupuncture experienced a reduction in symptom severity, in contrast to 63% of those undergoing pharmacological treatment, indicating that acupuncture was significantly more efficacious than either pharmacological therapy or no targeted intervention 121. While acupuncture demonstrated a positive impact in comparison to standard medical treatments for overall IBS symptoms, there was no evidence indicating that acupuncture improved symptom severity or quality of life when compared to sham acupuncture 121-123.

Acupuncture showed promising benefits in improving IBS symptoms and quality of life. A two-arm pragmatic RCT with 233 IBS patients who were an average duration of 13 years found that compared with usual care, acupuncture treatment significantly reduced the IBS Symptom Severity Score (IBS-SSS) at 3 months 124. Furthermore, this benefit largely persisted at 6-, 9- and 12-month, but disappeared at 24 months post-randomization 125. Meanwhile, the therapeutic effects of acupuncture even exceeded pharmacological treatment. A multicenter RCT in China enrolled 531 different-subtype IBS patients who were randomly allocated in a 2:1 ratio to either acupuncture or polyethylene glycol 4000 for IBS-C, or pinaverium bromide for IBS-D. This study found that the IBS-SSS separately decreased by 123.51 and 94.73 after acupuncture and drug treatment, with effects lasting up to 12 weeks, indicating that acupuncture may be more effective than pharmacological treatment in all the subtypes of IBS 126. Nonetheless, none of the trials that included a sham control demonstrated any advantages of acupuncture compared to sham acupuncture concerning IBS symptoms, overall quality of life, and even aspects of sleep quality 127-129. The design of trials for IBS treatments, as outlined by the Rome criteria, highlights that placebo effects pose a significant challenge, particularly when the endpoints evaluated are subjective. Consequently, it remains uncertain whether any genuine biological effects of authentic acupuncture could be obscured or masked by the substantial placebo effects associated with sham acupuncture.

### 7.2 Chronic functional constipation

Chronic constipation is prevalent globally, affecting around 14% of the population, and imposes a considerable economic strain due to healthcare expenses and its impact on patients' daily activities 130. Among the various subtypes of chronic constipation, CFC is the most frequently occurring. A recent survey employing the current Rome IV diagnostic questionnaire 131 indicated that the prevalence of chronic constipation is roughly 9%, with CFC representing about 6%, while the remaining 3% is equally divided between IBS-C and constipation resulting from opioid use. Therefore, CFC is a major public health problem and has an especially large effect on health. Traditionally, laxatives have been the primary pharmacological approach for chronic constipation due to their availability, affordability, and tolerance by most patients. However, this method can lead to side effects that vary with dosage, including bloating, gas, and diarrhea 130. Additionally, chronic functional constipation (CFC) is more frequent among women and older adults, many of whom express a willingness to explore acupuncture even with limited understanding of the practice 132. Therefore, an increasing number of CFC patients are integrating acupuncture into their existing healthcare plans.

The promising efficacy of acupuncture at increasing spontaneous gastrointestinal movements or motility in CFC patients was evaluated by following systemic reviews and meta-analyses (Table S4). A systematic review and meta-analysis with 9 RCTs found that compared with the medicine-treated group, EA significantly improved the frequency of spontaneous gastrointestinal movements, total response rates and reductions in symptoms score, indicating that EA was more effective and safer than medication in CFC patients 133, 134. Concomitantly, non-pharmacological treatments are becoming popular among patients with CFC. Another meta-analysis including 33 trials with 4342 participants examined the comparative effectiveness of non-pharmacological conservatives in treating CFC 135. This review demonstrated that compared with placebo interventions, probiotics, and other non-pharmacological treatments, acupuncture had a larger therapeutic effect on stool frequency, response rate, and had a lower rate of adverse events, providing some reasonable convincing evidence on acupuncture relatively ranked the best in managing CFC. Specifically, a comprehensive meta-analysis incorporating 6 studies with a total of 1,457 participants indicated that EA produced beneficial outcomes for severe CFC, with more extended treatment periods resulting in more pronounced effects 136. In summary, acupuncture is a promising non-pharmacological treatment for CFC, showing effects that are comparable or even exceed other first-line treatments of CFC with rare undesirable side effects.

Acupoint stimulation is more effective in alleviating constipation compared with sham acupuncture. A multicenter study involving 1,075 participants demonstrated that, in contrast to sham acupuncture, EA significantly enhanced the frequency of complete spontaneous bowel movements (CSBM) on a weekly basis and bettered the quality of life for patients experiencing severe CFC over the course of the 8-week treatment period; these benefits continued throughout the 12-week follow-up 137. Similarly, in a comparable RCT involving 30 individuals with CFC, the effectiveness of acupuncture was evaluated against that of sham acupuncture 138. Results indicated that acupuncture produced significant improvements, particularly in the frequency of CSBM, exceeding three times a week, with these enhancements persisting for four weeks following the treatment. It is important to highlight that, in comparison to pharmacological therapies, acupuncture proved to be at least equally effective or potentially more appropriate, exhibiting a minimal incidence of side effects for CFC. A multicenter, randomized, noninferiority study involving 560 participants aimed to compare EA and prucalopride in addressing severe chronic constipation. The results indicated that EA demonstrated noninferiority to prucalopride in alleviating weekly CSBM from weeks 3 to 8, while maintaining a favorable safety profile 139. Particularly, the 8-week EA effects could remain effective for 24 weeks following the treatment, indicating that acupuncture might be recommended as a valuable and promising new therapeutic option for patients with CFC. In a similar vein, another study indicated that both EA and mosapride significantly enhanced weekly CSBM, stool consistency, and difficulties associated with defecation. Nonetheless, acupuncture demonstrated greater efficacy in enhancing quality of life, as well as reducing depression and anxiety 140. Thus, acupuncture could serve as a potentially effective standalone treatment for patients experiencing CFC.

## 8. Limitations and implications for clinical research for acupuncture

Although acupuncture holds potential as a therapeutic intervention for gastrointestinal dysmotility, it cannot currently be recommended for clinical practice 141. Several systematic reviews have failed to recommend acupuncture for conditions such as IBS, gastroparesis, FD and others 88, 122, 142, primarily due to the high risk of bias present in most trials. Indeed, the poor quality of clinical trials is the existing problem. The Rome criteria for the design of IBS, FD and others treatment trials note that placebo effects present a particular challenge when the endpoints are subjective 143, 144. Consequently, researchers should carefully manage factors such as patient expectations for improvement, patient preferences, and non-specific therapeutic factors in acupuncture treatment 145. Despite the implementation of rigorously designed trials, accurately assessing the efficacy of acupuncture in IBS remains challenging due to the inherent complexities and potential biases associated with both comparative effectiveness and sham acupuncture control designs 122. These complexities complicate the evaluation of subjective, patient-reported outcomes, which are frequently utilized in IBS trials. This serves as the primary rationale for why acupuncture and sham acupuncture often yield similar improvements in IBS patients. Furthermore, scientific outcome assessments should not only incorporate a range of scales, but also consider objective indicators such as autonomic nervous system (ANS) activity and neurotransmitter levels, as these may elucidate underlying mechanisms of acupuncture in gastrointestinal dysmotility. Ultimately, conducting regular follow-up assessments after treatment to verify the long-term efficacy of acupuncture will contribute to generating more robust and convincing evidence in support of the study's findings.

## 9. Conclusion and prospects

Here, we argued that knowledge about the degree to which the autonomic nervous system is involved in maintaining the digestive tract's homeostasis is evolving quickly and will offer fresh perspectives on acupuncture from a treatment standpoint. The clinical research overall suggested that acupuncture could be a beneficial therapy for relieving gastrointestinal dysmotility, affecting areas from the esophagus to the colon, which encompasses conditions such as gastroparesis, FD, IBS, POI, and CFC. Mechanistically, studies with animal models and patients demonstrated that acupuncture, as a non-invasive strategy for somatosensory neuron stimulation, has potential to ameliorate gastrointestinal dysmotility via directly modulating ANS activation. Meanwhile, acupuncture suppressed inflammatory response and altered gastrointestinal hormones secreting via different somatic autonomic reflex pathways. Importantly, the therapeutic outcomes of acupuncture are shaped by, and even reliant on, selected acupoints, the frequency and intensity of stimulation, the type of gastrointestinal conditions, and the treatment frequency. Consequently, it is inaccurate to strictly categorize abdominal and hindlimb acupoints solely as triggers for sympathetic or vagal nerve activation that either enhances or diminishes gastrointestinal motility.

Significant advancements have been achieved in understanding the influence of the CNS on the gastrointestinal tract; however, several challenges and knowledge gaps persist in exploring how various regions of the CNS interact with gastrointestinal homeostatic functions and the effects of acupuncture stimulation. Symptoms of FD, IBS, gastroparesis, and CFC were overlapping and therapeutic acupoints were also similar, indicating that CNS exhibits a remarkable degree of plasticity and adaptation in gastrointestinal tract. The DMV-C-i neurons are distinct from the DMV-C-e neurons and various segments of the gastrointestinal tract could be influenced by unique sub-circuits of the GIVMC and GEVMC (Figure 3). In contrast to the relatively extensive information regarding the NTS and DMV, less information is available regarding the higher CNS nuclei involved in gastrointestinal tract, such as PVN and arcuate nucleus, particularly sympathetic associated brain regions. The central autonomic network is a sophisticated system that encompasses numerous feedback loops, both positive and negative, which regulate sympathetic and parasympathetic outputs. Consequently, predicting the ultimate outcomes of this system's activity is challenging, as prefrontal activation can lead to increased sympathetic activity in one scenario while promoting vagal activity in another. Nonetheless, the emergence and ongoing development of imaging, optogenetic, and various other methodologies facilitate the targeted activation or inhibition of particular neuronal subpopulations or pathways, which will contribute to uncovering the cellular mechanisms that govern the central control, regulation, and coordination of gastrointestinal functionality in upcoming studies. Additionally, a developing field is how alterations in the gut microbiome and particular infections are likely to impact innate immune disruptions, which may offer a reasonable rationale for certain gastrointestinal dysmotility issues, while not accounting for all cases, as well as new potential treatment avenues. Consequently, targeting the gut microbiome could be a valuable focus in acupuncture therapies, especially concerning stimulation of abdominal acupoints.

## Supplementary Material

Supplementary tables.
