# Supplementary material for: The Autonomic Nervous System in Acupuncture for Gastrointestinal Dysmotility: From Anatomical Insights to Clinical Medicine
Source: Int J Med Sci. 2025 May 20;22(11):2620–36. doi: 10.7150/ijms.107643 (PMC12163389; doi:10.7150/ijms.107643)
Supplement: Supplementary file 1 — Supplementary tables. [file ijmsv22p2620s1.pdf]

Table S1. Effect of acupuncture on gastroparesis

Year	Type of gastroparesis	RCTs	Sample size	Statistic value	Conclusion	Reference
2013	diabetic gastroparesis	14	948	P<0.00001	Acupuncture had a higher response rate and a more effective on improving dyspeptic symptoms including nausea, vomiting, loss of appetite, and bloating, but not gastric emptying.	24206922
2014	postoperative gastroparesis	7	370	P<0.0001	Acupuncture and acupuncture combined with medication showed significant higher total effective rate than control (usual care/medication)	25146082
2018	gastroparesis	32	2601	RR 1.22; 95%CI 1.16 to 1.28, I^2=0%	Acupuncture might have potential to improve the symptoms of gastroparesis, but the certain evidence was very low.	30560568

Table S2. Effect of acupuncture on postoperative ileus

Year	Type of gastroparesis	RCTs	Sample size	Statistic value	Conclusion	Reference
2014	cancer patients	33	/	/	The effects of acupuncture on different cancer-related aspects were shown, including postoperative urinary retention, quality of life, vasomotor syndrome, postoperative gastrointestinal dysfunction, prevention of prolonged postoperative ileus.	24338183
2017	POI	10	776	P<0.05	Compared with control groups, acupuncture was associated with shorter time to first flatus and time to first defecation. A subgroup analysis revealed that manual acupuncture was more effective on the time to first flatus and the time to first defecation; electroacupuncture was better in reducing the length of hospital stay.	29422935
2018	POI	22	1628	P<0.05	Acupuncture might have potential to reduce improve recovery of gastrointestinal function.	30151019
2019	POI	15	965	P<0.05	Meta-analysis results favored acupuncture treatment for POI by analysis of time to first flatus, time to first defecation, time to bowel sound recovery, time to first oral feeding and length of hospital stay.	31494334

Table S3. Effect of acupuncture on irritable bowel syndrome

Year	Type of gastroparesis	RCTs	Sample size	Statistic value	Conclusion	Reference
2012	IBS	17	1806	RR 1.28; 95%CI 1.12 to 1.45	Sham-controlled RCTs have found no benefits of acupuncture relative to a credible sham acupuncture control on IBS symptom severity or IBS-related quality of life. However, patients reported greater benefits from acupuncture than from pharmacological therapies.	22488079
2012	IBS	5	449	RR 1.28; 95%CI 1.12 to 1.45	Eighty-four per cent of patients in the acupuncture group had improvement in symptom severity compared to 63% of patients in the pharmacological treatment group (RR 1.28, 95% CI 1.12 to 1.45; 5 studies, 449 patients).	22592702
2014	IBS	6	664	P=0.001	Acupuncture exhibits clinically and statistically significant control of IBS symptoms.	24587665
2019	IBS-D	21	1834	P<0.05	(1) acupuncture combined with Chinese herbal medicine might result in more favorable improvements compared with the control group; (2) the combined method could markedly enhance the clinical efficacy in the meantime of remarkably reducing the scores of abdominal pain, abdominal distention/discomfort, diarrhea, diet condition, physical strength, and sleep quality compared with those in the matched groups treated with western medicine, or western medicine combined with Chinese herbal medicine.	31110553
2019	IBS	41	3440	P=0.04	Acupuncture was more effective relative to western medicine in alleviating IBS symptoms, whose effect might last 3 months. However, no significant difference was found when acupuncture was compared with sham acupuncture, in terms of effects on IBS symptoms and quality of life.	31814859
2020	IBS-D and FD	31	3234	P<0.05	Acupuncture produced more significant effect than drug related to the total symptom score,	33299403

2021	IBS	11	1509	P<0.05	IBS quality of life, recurrence rate, and IBS Symptom Severity Scale. Body-directed therapies (acupuncture and osteopathic medicine) showed a beneficial effect compared with standard medical treatment for overall IBS symptoms at 6 months follow-up, while no study found any difference between body-directed and sham therapies for abdominal pain or overall IBS symptoms.	34790245

Table S4. Effect of acupuncture on chronic functional constipation

Year	Type of gastroparesis	RCTs	Sample size	Statistic value	Conclusion	Reference
2013	CFC	15	1256	/	Acupuncture for CFC was probably as effective as conventional medical therapy in the change of bowel movements. Meanwhile, acupuncture for CFC was safe and may improve weekly spontaneous bowel movements, quality of life, and relevant symptoms.	23895148
2017	CFC	9	/	P<0.05	compared with the medicine-treated group, EA significantly improved the frequency of spontaneous gastrointestinal movements, total response rates and reductions in symptoms score, indicating that EA was more effective and safer than medication in CFC patients.	28630049
2019	CFC	33	4324	P<0.05	Compared with placebo interventions, probiotic, and other non-pharmacological treatments, acupuncture had larger therapeutic effect on stool frequency, response rate, and had lower rate of adverse events, providing some reasonable convincing evidences on acupuncture relatively ranked the best in managing CFT.	30125427
2020	severe CFC	6	1457	P<0.05	EA had favorable effects on CSFC, and the longer the treatment duration was, the better was the effect, but the effect showed a certain period of validity. However, the results may be influenced by the clinical heterogeneity of acupuncture points, depth of needling, intensity, and frequency of EA.	33183657
